# RNAi-Mediated Knockdown of Imaginal Disc Growth Factors (IDGFs) Genes Causes Developmental Malformation and Mortality in Melon Fly, *Zeugodacus cucurbitae*

**DOI:** 10.3389/fgene.2021.691382

**Published:** 2021-07-05

**Authors:** Shakil Ahmad, Momana Jamil, Muhammad Fahim, Shujing Zhang, Farman Ullah, Baoqian Lyu, Yanping Luo

**Affiliations:** ^1^School of Plant Protection, Hainan University, Haikou, China; ^2^Centre for Omic Sciences, Islamia College University, Peshawar, Pakistan; ^3^Department of Entomology, College of Plant Protection, China Agricultural University, Beijing, China; ^4^Environment and Plant Protection Institute, Chinese Academy of Tropical Agricultural Sciences/Key Laboratory of Integrated Pest Management on Tropical Crops, Ministry of Agriculture and Rural Affairs, Haikou, China

**Keywords:** chitinase, mortality, RNA interference, wings malformation, Tephritidae

## Abstract

This study reports the first successful use of oral feeding dsRNA technique for functional characterization of imaginal disc growth factors (IDGFs) genes (*IDGF1*, *IDGF3_1*, *IDGF4_0*, *IDGF4_1*, and *IDGF6*) in melon fly *Zeugodacus cucurbitae.* Phylogenetic and domain analysis indicates that these genes had high similarity with other Tephritidae fruit flies homolog and contain only one conserved domain among these five genes, which is glyco-18 domain (glyco-hydro-18 domain). Gene expression analysis at different developmental stages revealed that these genes were expressed at larval, pupal, and adult stages. To understand their role in different developmental stages, larvae were fed dsRNA-corresponding to each of the five IDGFs, in an artificial diet. RNAi-mediated knockdown of *IDGF1* shows no phenotypic effects but caused mortality (10.4%), while *IDGF4_0* caused malformed pharate at the adult stage where insects failed to shed their old cuticle and remained attached with their body, highest mortality (49.2%) was recorded compared to dsRNA-green fluorescent protein (GFP) or DEPC. Silencing of *IDGF3_1* and *IDGF4_1* cause lethal phenotype in larvae, (17.2%) and (40%) mortality was indexed in *Z. cucurbitae*. *IDGF6* was mainly expressed in pupae and adult stages, and its silencing caused a malformation in adult wings. The developmental defects such as malformation in wings, larval–larval lethality, pupal–adult malformation, and small body size show that IDGFs are key developmental genes in the melon fly. Our results provide a baseline for the melon fly management and understanding of IDGFs specific functions in *Z. cucurbitae.*

## Introduction

RNA interference (RNAi) was simultaneously discovered as a tool for functional genomics (Fire et al., 1998) and antiviral resistance strategy (Waterhouse et al., 1998). Since then, it has been explored and applied as an effective tool for the control of aphids (Zhao et al., 2018; Tariq et al., 2019; Ullah et al., 2020b), whiteflies (Grover et al., 2019), beetles (Mehlhorn et al., 2020), and lepidopterans pests (Rana et al., 2020), etc. Because of RNAi’s robustness and target precision, it has lowered pesticide pressure on humans and the atmosphere while minimizing negative effects on non-target and beneficial insects. Furthermore, RNAi knockdown and knock-out variants have opened new avenues in reverse genetics for functional characterization of previously uncharacterized genes. Numerous studies on RNAi use for transgenic insect resistance have been reported, either in cellular cytoplasm (Chung et al., 2021) or Chloroplast (Bally et al., 2018). Moreover, exogenous application of dsRNA is effective against herbivorous insect pests, both in the laboratory (San Miguel and Scott, 2016) and in field trials (Mehlhorn et al., 2020). Additionally, RNAi also has revolutionized sterile insect technique (SIT) through the use of dsRNAs targeted at genes involved in fertility or fecundity of insect pests (Darrington et al., 2017; Ullah et al., 2020b). However, the selection of efficient target genes for RNAi-mediated control strategy remains the pivotal player in the overall success and efficacy (Scott et al., 2013; Xu et al., 2016). In insects, the epithelial apical extracellular matrix (ECM) contains many fibrous proteins and polysaccharides synthesized or transmembrane, whose composition differs significantly, from insect chitinase to plants cellulose (Cosgrove, 2005; Öztürk-Çolak et al., 2016; Vuong-Brender et al., 2017). Exoskeleton is essential for epithelial barrier formation, maintaining body shape, homeostasis, and protect the insect from coming in contact with agrochemical, predators, and parasitoids (Galko and Krasnow, 2004; Yoshiyama et al., 2006; Turner, 2009; Shibata et al., 2010; Uv and Moussian, 2010; Jaspers et al., 2014). Many studies recently reported that ECM helps in the shaping of different organs, like *Drosophila* wings (Fernandes et al., 2010) and provide structural support to delicate internal organs but also protects them against damage caused by various environmental factors and microorganisms (Dittmer et al., 2015; Mun et al., 2015).

Various genes involved in cuticular synthesis and maintenance have been characterized (Pan et al., 2011). Among these, imaginal disc growth factors (IDGFs), which belong to Chitinase glycoside hydrolase 18 (GH18) family, are associated with insect’s molting and cuticle maintenance (Zhao et al., 2020). IDGFs were first identified from *Drosophila* imaginal disc cell cultures by fractionating conditioned medium (Kawamura et al., 1999; Zhu et al., 2008). IDGFs were confirmed to be the proteins cooperating with insulin that promote cell lineages derived from imaginal discs in *Drosophila melanogaster* (Kawamura et al., 1999; Varela et al., 2002; Zurovcová and Ayala, 2002). RNAi has been widely used to find out the functions of vital genes in different insects of economic importance (Tomoyasu and Denell, 2004; Chen et al., 2008; Gong et al., 2012; Asokan et al., 2013; Zhang et al., 2013; Qi et al., 2015; Wang et al., 2017; Ullah et al., 2020a). Recently, a study reported that silencing of *IDGF6* in *Bactrocera correcta* through RNAi significantly decreases the expression of *IDGF6*, causes larval mortality and wing malformation in adult flies (Zhao et al., 2020). Similar reports using RNAi techniques for silencing essential genes were recorded in severe phenotypes abnormalities in different insect species (Zhu et al., 2008; Bellés, 2010; Scott et al., 2013; Xi et al., 2015). Although in model insects *D. melanogaster*, IDGFs have been reported systematically, and specific functional information in *Zeugodacus cucurbitae* are still unknown. In *Drosophila*, these five non-enzymatic IDGFs (*IDGF1*, *IDGF3_1*, *IDGF4_0*, *IDGF4_1*, and *IDGF6*) are involved in the maintenance of ECM scaffold against chitinolytic degradation, and plays a vital role in physiological processes such as adult eclosion, development regulation, and blood sugar reduction of insects (Galko and Krasnow, 2004). Among these genes, the function of the *IDGF4* gene has been recently described in the defense barrier and development of *Bactrocera dorsalis* (Diptera: Tephritidae) (Gu et al., 2019). However, very little information is available on the rest of the member genes. Targeting genes involved in cuticular formation may provide an effective way for pest control.

Melon fly, *Z. cucurbitae* Coquillett (Diptera: Tephritidae) is one of the most destructive pests that cause severe economic loss to cucurbit crops (Gogi et al., 2009). Different researchers reported its losses in various crops to range up to 30–100% (Dhillon et al., 2005; Subedi et al., 2021). Researchers reported many strategies to control fruit flies which includes pheromones (Shelly et al., 2004; Panhwar, 2005), cultural practices (Gogi et al., 2007, 2009), biological controls (Drew et al., 2003), lure mixtures (Vargas et al., 2008, 2010), and hot water treatment (Panhwar, 2005). Insecticide applications are less effective due to larvae developing and feeding inside the fruit, covered by fruit pulp, and not exposed to direct insecticides (Yee et al., 2007; Gogi et al., 2009; Sapkota et al., 2010). Also, insecticides contaminate the environment, have a deleterious impact on predators and parasitoids of insect pests, develop resistance, induces insect pest populations and have maximum residue levels (MRLs) issues (Desneux et al., 2007; Baig et al., 2009; Decourtye et al., 2013; Gebregergis, 2018; Jactel et al., 2019; Ullah et al., 2019a, b). Therefore, novel approaches such as RNAi will provide novel ways to control *Z. cucurbitae* and provide insight into functional genomics of the target genes in ECM formation.

In this paper, we cloned and identified full-length cDNA of five IDGF family genes from *Z. cucurbitae*, which are least characterized in Tehpritidae. We then analyzed gene expression patterns in eight different developmental stages of *Z. cucurbitae* using real-time quantitative PCR (RT-qPCR). dsRNA-mediated RNAi technology was applied to explore the function of five-member genes of IDGF family in *Z. cucurbitae* at larval and adult stages. Knockdown of *IDGF3_1*, *IDGF4_0*, *IDGF4_1*, and *IDGF6* genes led to various types of developmental defects and mortality except *IDGF1*, where the dsRNA treated larvae showed minimal mortality and no visible phenotypes. Our data provide a baseline for the role of IDGFs genes in developmental stages of *Z. cucurbitae* and identify the potential target for RNAi mediated pest control strategy.

## Materials and Methods

### Insects Rearing

Colony of *Z. cucurbitae* was reared for many generations in the insect rearing room at 25 ± 1°C and 75% relative humidity, with a 14:10 h (light: dark) photoperiod at Hainan University, Haikou, China. Larvae were fed with artificial food as described previously (Liu et al., 2020). Fruit flies were reared on a ratio of 3:1 of sugar and yeast for around 10–12 generations in 45 cm × 45 cm × 50 cm cages before the experiment to eradicate local environmental impact.

### Cloning of IDGFs Genes

To detect the expression pattern of five different genes (*IDGF1*, *IDGF3_1*, *IDGF4_0*, *IDGF4_1*, and *IDGF6*), total RNA was isolated from eight different developmental stages of *Z. cucurbitae*. Briefly, A total of ten individuals according to the body size (Per replication: L2 20 larvae, L3-1 10 larvae, L3-2 10 larvae, P-E, P-M, P-L 5 pupae for each, A-E and A-M 2 adults for each) were randomly collected and mixed for RNA extraction. cDNA was synthesized using commercially available HiScript^®^ III 1st Strand cDNA Synthesis Kit following the manufacturer’s instructions. RT-qPCR was performed to verify IDGFs gene fragment (Supplementary Table 3) from *Z. cucurbitae* using Prime STAR^®^ HS DNA Polymerase (Takara, Japan) under the following conditions: initial denaturation at 94°C for 5 min; followed by 30 cycles of Denaturation at 94°C for 30 s, annealing at 58°C for 30 s, extension at 72°C (according to the size of each gene) and final extension at 72°C for 5 min. Amplified products were examined by 1.2% agarose gel electrophoresis and purified using a Universal DNA Purification kit (Tiangen, China). Amplified PCR products were cloned into a pMD^TM^18-T vector (Takara, Japan), and verified by sequencing at Sangon Biotech Company Shanghai, China.

### Phylogenetic Analysis

We used MEGA 6.0 software to construct a phylogenetic tree through the maximum likelihood method JTT matrix-based model with 1,000 replications of bootstrap analysis (Tamura et al., 2013). The full name of species used in this tree construction and the short names used are all listed along with GenBank accession numbers in Supplementary Table 1.

### dsRNA Preparation and Feeding

dsRNA was synthesized using T7 RiboMAX^TM^ Express RNAi System (Promega, United States). Each primer used for PCR contained a 5′ T7RNA polymerase binding site (GAATTAATACGACTCACTATAGGGAGA) followed by the sequence-specific for the target gene i.e., *IDGF1*, *IDGF3_1*, *IDGF4_0*, *IDGF4_1*, and *IDGF6* (Supplementary Table 3). These primers were used to amplify the template for the synthesis of forward and reverse RNA. dsRNA was purified according to manufacturer’s instructions and the integrity and quantities of all synthesized dsRNA products were determined by 1.2% agarose gel electrophoresis. Their concentration was measured using the NanoDrop2000 spectrophotometer. dsRNA of green fluorescent protein (GFP) and DEPC was used as a negative control. To investigate the biological functions of each chitinase gene of *Z. cucurbitae*, dsRNA was fed to 2 days old third instar larvae for 48 h and then shifted to the new food contain dsRNA for another 48 h. Five biological replications were performed with sixty individuals in each replicate. Each replicates fed with 6 g artificial food contained 60 μl dsRNA (1,000 ng/μl), dsGFP, and DEPC. Larval body size, mortality, and phenotype were examined 24 h post-feeding at each developmental stage till the adult’s sexual maturity.

### Detection of Gene Expression by RT-qPCR

To understand the temporal gene expression profile of *IDGF1*, *IDGF3_1*, *IDGF4_0*, *IDGF4_1*, and *IDGF6* of *Z. cucurbitae*, RT-qPCR was performed at different developmental stages. RT-qPCR was performed using SYBR^®^ Premix Ex Taq^TM^ II (TliRNaseH Plus) (Takara, Japan) on an ABI 7500 instrument (United States). The PCR reaction includes 10 μl SYBER Green mix, 1 μl cDNA, 1 μl each of forward and reverse primers and 7 μl of ddH_2_O with three technical and three biological replicates for each gene expression. The elongation factor 1 alpha (*EF1*α) was used as endogenous reference genes for data normalization, and a relative transcript level of IDGFs was calculated with the 2^–ΔΔCt^ method (Livak and Schmittgen, 2001). All the primers used in this study are shown in Supplementary Table 3.

### Silencing of Chitinase Genes of *Zeugodacus cucurbitae*

To observe phenotype, third early-instar larvae (2 days old) was fed with 6 g food mixed with 60 μl dsRNA or dsGFP (1,000 ng/μl) or DEPC for 48 h and transferred to a new artificial diet with the same treatment for another 48 h. After 96 h, larvae were shifted to soil for pupation. Two individuals from each replication of each group were killed every 24 h until the pupal stage to determine RNAi efficiency, while the others continued to feed. Similarly, two individuals were killed at the adult stage (24 h old), to test the RNAi efficiency. The stability of dsRNAs in the artificial diet, 1 g of each diet was collected 24 h post-feeding. The artificial diet was diluted in 50 μl distilled water, and the dsRNAs were observed in 1% agarose gel electrophoresis. Mortality was recorded by counting the flies number in each group after 24 h. The phenotype effects were observed in each developmental stage until 10 days of the adult’s emergence.

### Statistical Analysis

Statistical analysis was performed to measure the significant differences between each different group. Chitinase-like protein expression was quantified in the larvae, Pupae, and adults treated with dsRNA-GFP, DEPC, and gene-specific dsRNA. Statistical significance of differences in gene expression levels among samples was assessed using one-way ANOVA with a 0.05 level of significance (95% confidence interval) GraphPad Prism 8.01 for Windows (GraphPad Software, San Diego, CA, United States)^1^.

## Results

### Characterization and Phylogenetic Analysis of IDGFs of *Zeugodacus cucurbitae*

**FIGURE 1:**
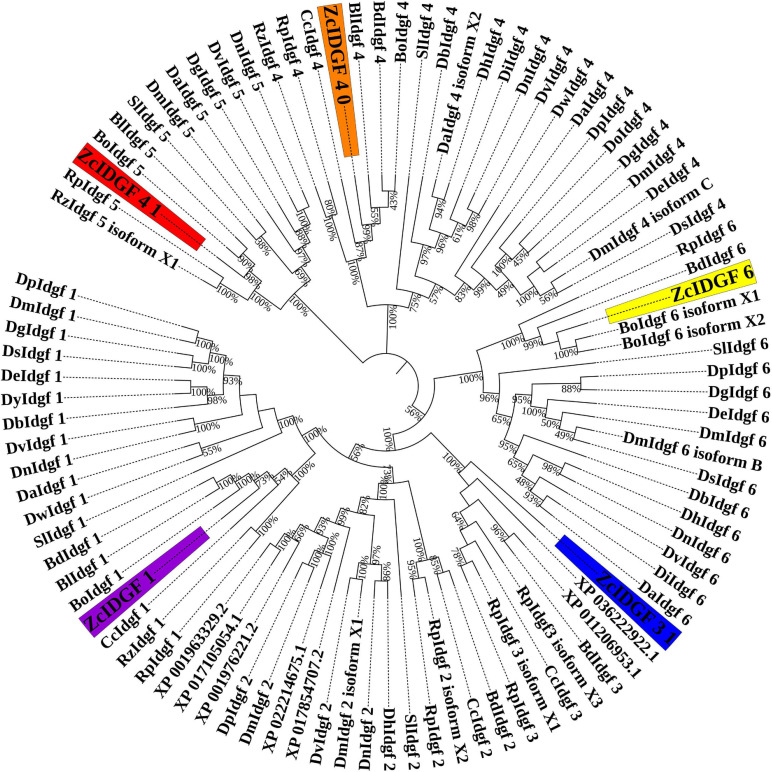
Phylogenetic analysis of IDGFs genes with model family Tephritidae (taxid: 7211) and Drosophilidae (taxid: 7214) are shown in the tree. The highlighted part indicates our target genes. Tree indicates relationship between IDGFs gene and species tree. Maximum likelihood method was used to construct insects IDGFs coding sequences phylogenetic tree. Complete details of all IDGFs are listed in Supplementary Table 1.

Imaginal disc growth factors genes (*IDGF1*, *IDGF3_1*, *IDGF4_0*, *IDGF4_1*, and *IDGF6*) were cloned from *Z. cucurbitae* (Supplementary Table 2). They were compared with IDGF genes with Tephritidae (taxid: 7211) and Drosophilidae (taxid: 7214) as a model family (Supplementary Table 1). The five IDGF genes were highly conserved and had high homology with members of Tephritidae than Drosophilidae (Figure 1).

Nucleotide sequence analysis shows that *IDGF1* of *Z. cucurbitae* had the maximum similarity with a homolog *Bison latifrons* and *B. dorsalis* (92%) followed by *Bactrocera oleae* (91%) and *Ceratitis capitata* (89%). Compared with similar in *Drosophila*, the highest identity was recorded with *Drosophila virilis* (69%). *IDGF3_1* shows highest similarity with *B. dorsalis* and *B. latifrons* (94%) followed by *B. oleae* (93%) and *Rhagoletis pomonella* (91%). Compared with the similar Drosophilidae, the highest identity was revealed with *Drosophila hydei* and *D. virilis* (71%). For *IDGF4_0*, the maximum similarity was recorded with *B. latifrons* and *B. dorsalis* (98%), followed by *B. oleae* (96%) and *C. capitata* (92%). Compared to the similar in Drosophilidae, the highest identity of *IDGF4_0* revealed with *D. hydei* and *D. virilis* (83%). Nucleotide sequence analysis revealed that the *IDGF4_1* of *Z. cucurbitae* had highest identity with a homolog from *B. oleae* (79%), *B. latifrons* (76%), *C. capitata* (72%), followed by *Rhagoletis zephyria* and *R. pomonella* (71%). Compared to the same Drosophilidae, the highest identity of *IDGF4_1* revealed with *Drosophila mojavensis* (58%). Comparison of nucleotide sequence within Tephritidae family revealed that *IDGF6* of *Z. cucurbitae* has high homology with *B. dorsalis* (96%), *B. latifrons* (96%), followed by *B. oleae*, and *C. capitata* (94%). In the family Drosophilidae, the highest identity of *IDGF6* was observed with *D. melanogaster* (77%).

### Architectures of Domain and Catalytic Motif of IDGFs in *Zeugodacus cucurbitae*

**FIGURE 2:**
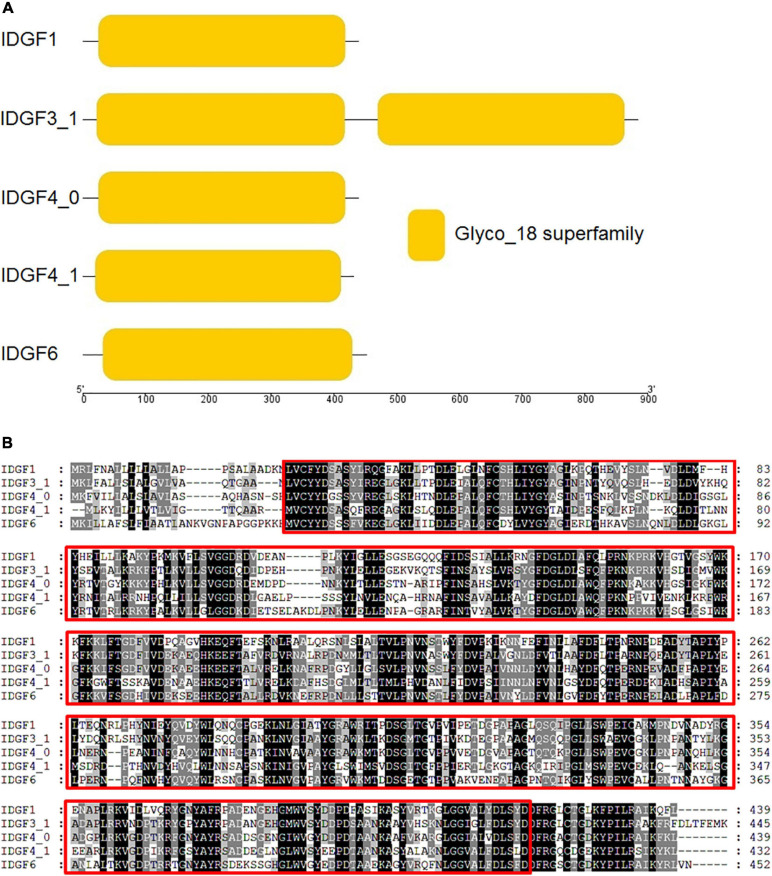
**(A)** Imaginal disc growth factors gene, also their domain architecture and motif in *Z. cucurbitae.* The deduced amino acid sequences were used to predict the domain architectures of the five IDGFs genes using online conserved domain database (CDD) and presented through TBTool software. **(B)** Amino acid sequence alignment of IDGFs was performed using ClustalW alignment method in MEGA7. In GeneDoc program, ClustalW alignment was used to shade the identical and similar amino acids in the alignment. The conserved regions among five IDGFs sequences are tinted with red box. Dark shade indicates identical amino acids and gray shade represents similar amino acids.

We used amino acid sequences of the five IDGFs genes, i.e., *IDGF1*, *IDGF3_1*, *IDGF4_0*, *IDGF4_1*, and *IDGF6*, for domain architectures using pfam online tool (Figure 2A). Our results show that all predicted amino acid sequences contained ≥ 1 Glyco_hydro_18 superfamily domain (PFAM accession: PF00704).

In particular, *IDGF3_1* had two copies of Glyco_hydro_18 superfamily domains, whereas the remaining amino acid sequences, *IDGF1*, *IDGF4_0*, *IDGF4_1*, and *IDGF6*, had only one copy. Sequence alignment showed that five IDGFs genes have well-conserved regions, including the specific sites for gene activity (Figure 2B). However, no chitin-binding domain (CBD) was found at the *C*-terminus. Further, *IDGF1* has two *N*-glycosylation sites at positions 208 and 220 in the N-terminal extracellular domain, while *IDGF3_1* has three potential *N*-glycosylation sites at positions 219, 665, and 791. The *IDGF4_0* has two *N*-glycosylation sites at positions 65 and 222, and *IDGF4_1* also has two potential *N*-glycosylation sites at positions 83 and 278 in the *N*-terminal extracellular domain. *IDGF6* has only one *N*-glycosylation site at position 233 (Supplementary Figure 1).

### Temporal Expression Patterns of IDGFs in *Zeugodacus cucurbitae* Wild-Type

**FIGURE 3:**
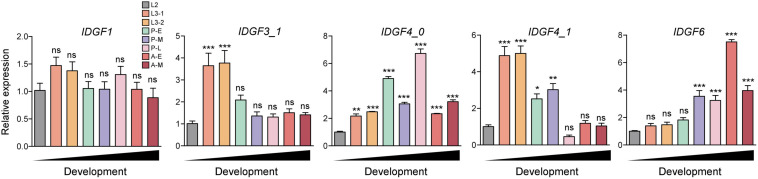
Temporal expression of eight developmental stages of *Z. cucurbitae* was determined, RNA was extracted from the whole body of flies in different developmental stages including 2nd instar larvae (L2), 3rd early-instar larvae (L3-1), Third late instar larvae (L3-3), 1–2 days mixed pupae as early pupae (P-E), 5–6 days as mid pupae (P-M), and 7–9 days as of late pupae (P-L), 1–2 days adults as (A-E), and 10-day adults as (A-M). We had presented our results after normalization against reference gene *EF*α*1* as the relative expression. All IDGFs gene expression is relative to the gene expression of each gene in 2nd instar larvae. One-way ANOVA with *post hoc* Tukey test was used to test the statistical significance *p < 0.05; **p < 0.01; ***p ≤ 0.001, ns: not significant.

Temporal expression of five IDGFs genes in eight different developmental stages of *Z. cucurbitae* was determined using qPCR. IDGFs genes varied expression in certain developmental stages (*t*-tests: *P* < 0.05). We observed that the expression of *IDGF1* slightly increased in early larval instars and almost tended to stabilize until the pupal stage. The *IDGF3* significantly increased in expression at the first two larval stages. *IDGF4_0* significantly expressed in all stages. *IDGF4_1* was significantly expressed in larval and mid-pupal stage. While *IDGF6* was strongly expressed in pupal and adult stages only (Figure 3). The expression pattern of IDGFs indicates their pivotal roles in different developmental stages.

### dsRNA-Mediated Knockdown of IDGFs Genes in *Zeugodacus cucurbitae*

RNAi technique has been used to inhibit target gene expression as a temporal knockdown strategy. Recently, RNAi techniques are being used in many studies for the management of different insects. *Z. cucurbitae* is an economically efficient fruit fly that causes a risk to many crop production and requires economically quarantine restrictions and eradication techniques. We developed a dsRNA feeding method for functional characterization of IDGF genes in *Z. cucurbitae* and identifying potential genes for effective management strategy. Compared to other strategies, dsRNA mixed with artificial food (Asimakis et al., 2019), is a non-invasive process and is less laborious in various systems, i.e., synthesized dsRNA (Turner et al., 2006), siRNA (Wuriyanghan et al., 2011), virus-derived RNA (Kumar et al., 2012), and transgenic hairpin RNA (Baum et al., 2007).

**FIGURE 4:**
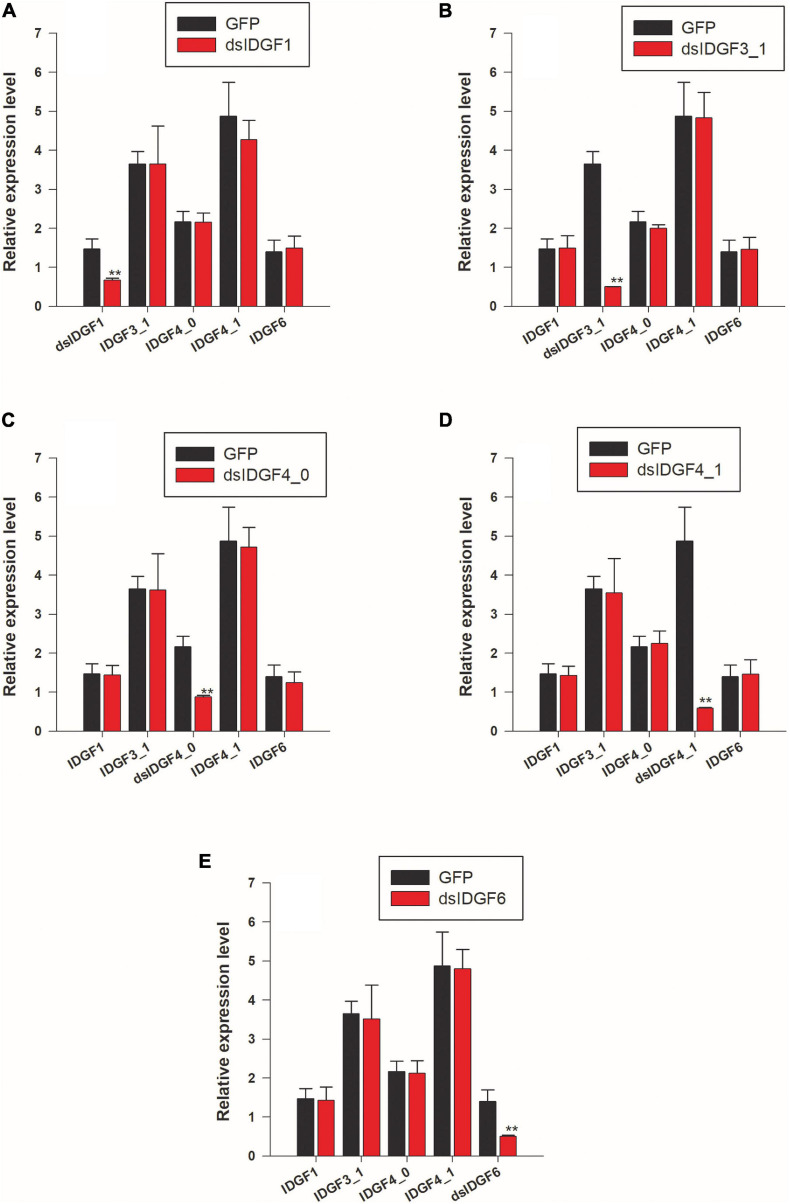
RNAi suppresses only the target transcripts. **(A)** Larvae fed with ds*IDGF1* and the other four genes are non-target transcript. **(B)** Larvae fed with ds*IDGF3_1.* **(C)** Larvae fed with ds*IDGF4_0*. **(D)** Larvae fed with ds*IDGF4_1*. **(E)** Larvae fed with ds*IDGF6*. No effects observed on non-target transcript.

In all functional studies, two control groups, i.e., dsRNA-GFP and DEPC were used with no difference among these two control groups as compared to wild-type, e.g., no malformed wings, no pupal–adult malformation, and no larval–larval lethality in both the control groups, indicating that these phenotype abnormalities were related to the dsRNA homology depended on IDGFs genes knockdown. After knockdown for each gene, the expression level for other genes was determined by qPCR, and no non-target effects were observed, which prove the effectiveness of RNAi in *Z. cucurbitae* (Figure 4).

### dsRNA-*IDGF1* Shows No Phenotypic Defects in *Zeugodacus cucurbitae*

Significant difference with a control group in the expression level of *IDGF1* was observed 24 h post-feeding of dsRNA-*IDGF1*, also a significant decrease in mRNA expression level was observed at 48, 72, 96, and 240 h. However, *IDGF1* knockdown causes (10.4%) mortality in *Z. cucurbitae.*

### *IDGF3_1* and *IDGF4_1* Contribute to the Larval–Larval Molt of *Zeugodacus cucurbitae*

**FIGURE 5:**
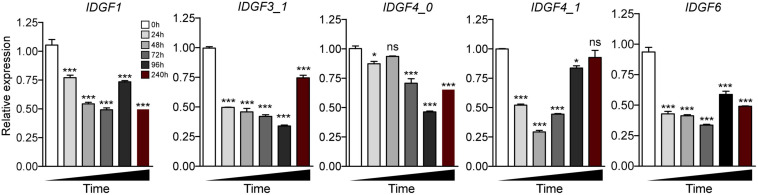
Relative expression pattern of IDGFs in different time intervals post feeding to dsRNA or dsGFP or DEPC were determined as mean (±SE) of the three biological replicates, and two flies were used per pooled RNA sample with control as the calibrator, i.e., cDNA from non-RNAi flies (only fed on artificial diet with DEPC-water and dsGFP). *EF1*α is used as the internal control. One-way ANOVA with post hoc Tukey test was used to test the statistical significance *p < 0.05; **p < 0.01; ***p ≤ 0.001, ns: not significant.

**FIGURE 6:**
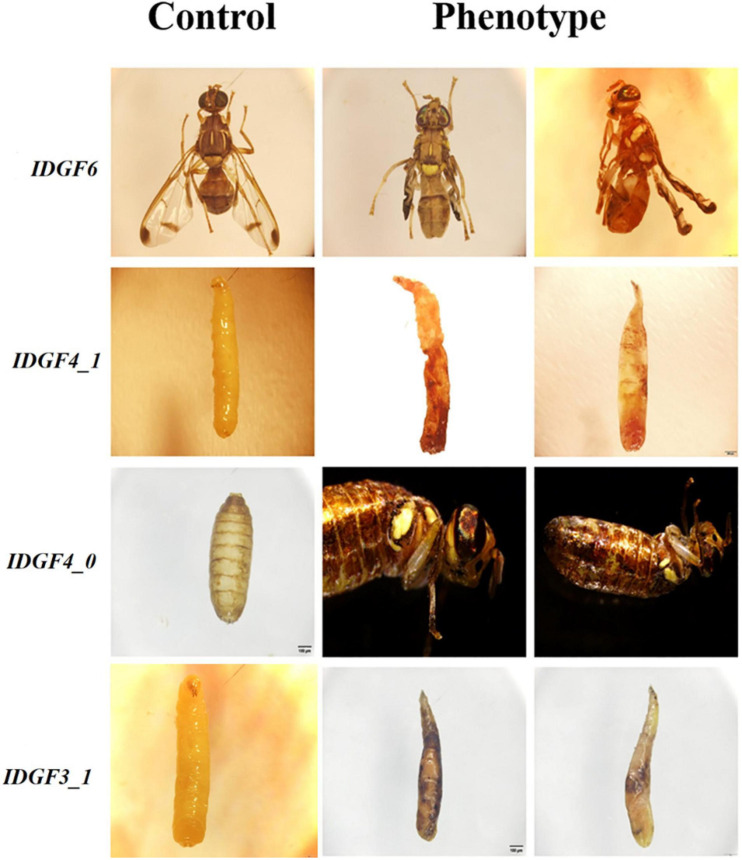
Phenotypes, abnormalities after feeding dsRNA of IDGFs compared to control group dsGFP or DEPC in different developmental stages of *Z. cucurbitae*. All Pictures were taken with a scale bar 200 μm. The Control group represents either dsGFP or DEPC, and the Phenotype group represents abnormalities post feeding dsRNA for each gene. In phenotypes groups *IDGF6* represents wings malformation in *Z. cucurbitae, IDGF3_1* and *IDGF4_1* represents larval lethal phenotypes and *IDGF4_0* represents phenotype at pupal–adult stage where flies fail to shed their old cuticle.

**FIGURE 7:**
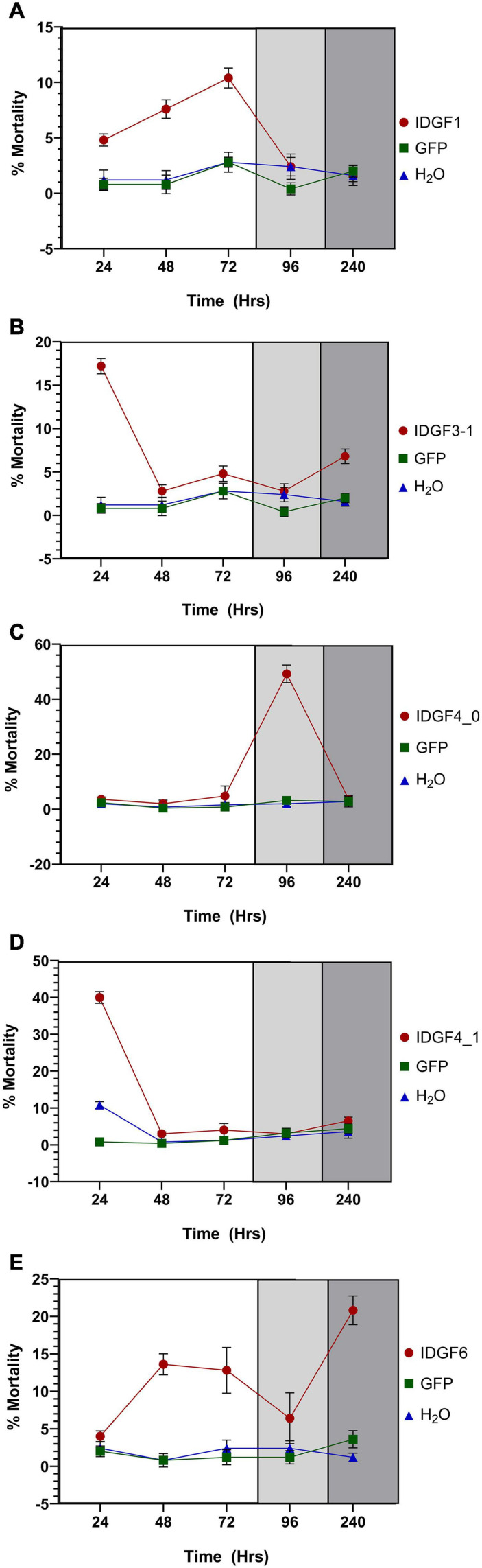
Mortality rate (%) of *Z. cucurbitae* at different developmental stages after being artificially fed with dsGFP or DEPC or dsRNA of IDGFs. The letters **(A–E)** represents *IDGF1*, *IDGF3_1*, *IDGF4_0*, *IDGF4_1*, and *IDGF6*. The white portion represent larval stages, light gray indicates pupal stage, and dark gray indicates adult stage of *Z*. *cucurbitae*. The values are presented as the mean (±SE) of five biological replications (50 insects were used per replicate). Treatments were compared using one-way ANOVA (Turkey’s test, *p* < 0.05).

Severe developmental defects and phenotypic abnormalities were observed when dsRNA-*IDGF3_1* or dsRNA-*IDGF4_1* were fed to the 2-day-old third instar larvae. Since these genes are highly expressed in the larval stage (Figure 3), therefore, the decrease in expression led to cuticular degradation in old larvae, resulting in the hindrance of larval molting (Figures 5, 6). After feeding dsRNA-*IDGF3_1*, the highest mortality recorded was (17.2%) at 24 h (Figure 7). The pupae size of dsRNA-*IDGF3_1* fed larvae reduced by 50% as compared to the control group. The remaining individuals completed metamorphosis into adults. Further, after feeding dsRNA-*IDGF4_1*, the highest mortality (40%) was recorded at 24 h compared to dsRNA-GFP and DEPC, and about (20%) individuals died and turned black with abnormal pigmentation. These results suggest that both *IDGF3_1* and *IDGF4_1* play an essential role in larval molting.

### *IDGF4_0* Is Required for Pupal–Adult Molt of *Zeugodacus cucurbitae*

Individuals fed with dsRNA-*IDGF4_0* exhibited phenotype at pharate adult stage as compared to the control group. After 5–6 days of pupation, a mortality of 49.2% was recorded (Figure 7). Furthermore, *Z. cucurbitae* failed to shed their old cuticle, and the mature cuticle was visible under the old cuticle resulting in the splitting of the old pronotal cuticle (Figure 6). In comparison, no abnormalities were recorded in control groups, either dsRNA-GFP or DEPC.

### *IDGF6* Is Required for Wings Formation of *Zeugodacus cucurbitae*

When dsRNA for *IDGF6* was fed to the third larval instar of *Z. cucurbitae* no phenotype was observed in larval or pupal stage. The larvae had completed the larval–larval and larval–pupal molts; however, there were some notable differences during the molts. The pupae usually contract their abdomens compared to control (dsRNA-GFP or DEPC) to the same extent. The adult’s eclosion was also the same as the control group. A remarkable phenotype was observed at the adult stage, where the wings were malformed and curled, which did not spread normally (Figure 6). **A**pproximately 90% of individuals with malformed wings died within 10 days of emergence. The highest mortality rate (20.8%) was recorded at 240 h post-feeding dsRNA-*IDGF6* compared to the control group (Figure 7). Moreover, no malformed wings were observed in the control group in dsRNA-GFP and DEPC, and all the flies lived normally.

## Discussion

Based on these results, we had applied the oral feeding dsRNA technique for the first time in melon fly *Z. cucurbitae* to know the specific function of IDGFs genes. IDGFs belong from a poorly described GH 18 Chitinase family with proteins without catalytic activity (Funkhouser and Aronson, 2007). Using five IDGFs genes (mentioned above) nucleotide sequences of Tephritidae, the Maximum likelihood method was applied to get a phylogenetic tree, which shows a high similarity with the homolog in other Tephritidae fruit flies (Figure 1 and Supplementary Table 1). Chitinase is known to degrade chitin to the low molecular weight Chit oligosaccharides and play an important role in the growth and development of insects (Zhu et al., 2016). The number of chitinase family genes in different insects ranges from 9 *Acyrthosiphon pisum* to 24 in *Tribolium castaneum* (Zhu et al., 2008; Arakane and Muthukrishnan, 2010; Nakabachi et al., 2010; Tetreau et al., 2015; Omar et al., 2019). Zhao et al. (2018) reported that plant-mediated RNAi of chitin synthase 1 (*CHS1*) gene in *Sitobion avenae* causes ∼50% decreased expression, whereas ∼20% reduction was observed in number of aphids and ecdysis. RNAi-mediated knockdown of *MpNav* gene expression caused up to 65% mortality in 3rd instar nymphs and lowered the longevity and fecundity in adult peach-potato aphid, *Myzus persicae* (Tariq et al., 2019). Oral-delivery-mediated RNAi of *CHS1* causes mortality and also disrupted the adult longevity and fecundity of the cotton-melon aphid, *Aphis gossypii* (Ullah et al., 2020b).

Temporal expression analysis in eight different developmental stages showed that these genes are highly expressed in different stages: larval–larval, larval–pupal, and pupal–adults, which indicate a vital role in the growth and development of these stages. *IDGF1* was expressed in all stages, mostly in larval stages, and it’s silencing caused mortality, but no phenotypic effects were observed. It would be an interesting study to compare the impact of IDGF family knockdown effect on the anatomy and histology of the melon fly. Furthermore, *IDGF3_1* and *IDGF4_1* were highly expressed in a larval stage, and silencing of both of these genes caused lethal phenotype in larvae (Figure 6) and caused mortality. Taken together, our results are consistent with few previous studies focused on IDGFs role in insect molting. A prior study on further vitro cell growth tests reported that combined with the insulin, *IDGF1* or *IDGF2* proteins stimulated the cultured imaginal disk cells growth (Hipfner and Cohen, 1999; Kawamura et al., 1999). Previously, it has been shown that *IDGF1* is expressed in the large salivary gland cells. Along with *IDGF3* its expression is lower as compared to *IDGF2* and *IDGF4* (Kawamura et al., 1999) *in vitro* cell growth tests combined with the insulin revealed that *IDGF1* or *IDGF2* proteins stimulated the cultured imaginal disk cells growth (Hipfner and Cohen, 1999; Kawamura et al., 1999). In a previous functional study of IDGFs, genes reported that individually *IDGF1* knocked down through RNAi in a model specie *Drosophila*, shows narrowed ECM thickness and displayed severe epidermal lesions in the larvae (Pesch et al., 2016). Similarly, expression levels of *IDGF3_1* after dsRNA feeding significantly decrease at 24, 48, 72, 96, and 240 h post-feeding. Pesch et al. (2016) found that in *Drosophila*, the IDGFs are essential for larval and adult molting. dsRNA-mediated silencing of IDGF family genes resulted in deformed cuticles, larval, and adult molting defects in *Drosophila*. Individual *IDGF3* knockdown via RNAi resulted in cuticle molting defects (Zurovcova et al., 2019). In similar studies, Espinoza and Berg (2020) found that overexpressing *IDGF3* leads to defects in the dorsal appendage with ∼50% frequency.

Individual knockdown of *IDGF4* in 3rd instar larvae through RNAi led to reduced larvae’s survival rate under high temperature and caused malformation as adults. This finding indicates the role of *IDGF4* in the defense barrier and development of fruit flies (Gu et al., 2019). Several studies have mainly focused on the function of *IDGF4* in larval stages, while only two related research articles were founded about another key developmental stage: pupae. In *T. castaneum*, when ds*IDGF4* injected either into penultimate or to the last instar larvae shows normal pupation but caused mortality during adult eclosion (Zhu et al., 2008). In *B. mori*, proteins with a decisively different expression profile among wild-type and scale-less wing mutants were verified and revealed that one *IDGF* gene was correlated to the differentiation of scale cells and development (Shi et al., 2013). Likewise, in homologs, specie *B. dorsalis*, dsRNA-*IDGF4* feeding in artificial food caused wings malformation and mortality (Gu et al., 2019). Furthermore, in *B. correcta*, dsRNA-*IDGF6* mediated strategy led to reduced gene expression of *IDGF6*, resulting in larval death and adult wing malformation. The knockdown of *IDGF6* led to decreased chitinase activity, resulting in stabilizing old cuticles and reduced body size (Zhao et al., 2020). Pesch et al., reported that *IDGF6* RNAi-induced mutants showed high mortality, and severe cuticle defects were observed in other mutants (Pesch et al., 2016). *IDGF6* is critical for larval cuticle barrier formation and protection against invasive microorganisms and mechanical stresses (Pesch et al., 2016). Therefore, *IDGF6* may prove to be an effective target for RNAi-based management.

In the current study, we observed differential responses to dsRNA uptake. For example, in *IDGF4_1*, the gene expression goes down in response to dsRNA feeding. However, the *IDGF4_1* expression recovers 48 h after dsRNA feeding. This phenomenon has been widely observed and attributed to various potential mechanisms, including the mutations of target genes or core RNAi machinery genes, enhanced dsRNA degradation, and lower dsRNA uptake (Zhu and Palli, 2020). For example, The Western Corn Cutworm (WCR) exhibited resistance to transgenic maize expressing *DvSnf7* dsRNA due to impaired luminal uptake. This resistance was not *DvSnf7* dsRNA specific, as indicated by cross-resistance to all other tested dsRNAs (Khajuria et al., 2018). The differential response of IDGF genes to the corresponding dsRNA may provide an excellent tool to further demystify the dsRNA resistance in insect pests. Overall, IDGFs can be used as potential target genes for pest control because of their function in different developmental stages. The malformation in wings, larval–larval lethality and pupal–adult malformation and small body size, and the highly conserved traits show that IDGFs are key genes for the pest. Furthermore, our results will pave the way for in-depth functional analysis of IDGFs family members and identify suitable insect control strategies through RNAi.

## Data Availability Statement

The original contributions presented in the study are included in the article/Supplementary Material, further inquiries can be directed to the corresponding authors.

## Author Contributions

SA and YL: designing research and funding acquisition. SA and MJ: methodology. SA, MF, and MJ: data curation and formal analysis. SA: performing research. SA, MF, FU, MJ, YL, BL, and SZ: writing – review and editing. YL and BL: supervision. All authors have read and agreed to the published version of the manuscript.

## Conflict of Interest

The authors declare that the research was conducted in the absence of any commercial or financial relationships that could be construed as a potential conflict of interest.

## Footnotes

1 www.graphpad.com
