# Supplementary material for: RNAi-Mediated Knockdown of Imaginal Disc Growth Factors (IDGFs) Genes Causes Developmental Malformation and Mortality in Melon Fly, Zeugodacus cucurbitae
Source: Front Genet. 2021 Jul 5;12:691382. doi: 10.3389/fgene.2021.691382 (PMC8287652; doi:10.3389/fgene.2021.691382)
Supplement: Supplementary file 1 [file Data_Sheet_1.docx]

| Table S1. Details of IDGFs protein sequences used for Phylogenetic analysis. | | | |
| --- | --- | --- | --- |
| No. | Species | Name used in P. tree | Accession Number |
| 1 | *Zeugodacus cucurbitae* | ZcIDGF 4_0 | XP_011185355.1 |
| 2 | *Bactrocera dorsalis* | BdIdgf 4 | XP_011213425.1 |
| 3 | *Bactrocera latifrons* | BlIdgf 4 | XP_018800655.1 |
| 4 | *Bactrocera oleae* | BoIdgf 4 | XP_014099295.1 |
| 5 | *Ceratitis capitata* | CcIdgf 4 | XP_004521633.1 |
| 6 | *Scaptodrosophila lebanonensis* | SlIdgf 4 | XP_030379402.1 |
| 7 | *Drosophila serrata* | DsIdgf 4 | XP_020802056.1 |
| 8 | *Drosophila obscura* | DoIdgf 4 | XP_022234159.1 |
| 9 | *Drosophila pseudoobscura* | DpIdgf 4 | XP_001354408.3 |
| 10 | *Drosophila persimilis* | DpIdgf 4 | XP_026846962.1 |
| 11 | *Drosophila guanche* | DgIdgf 4 | XP_034123648.1 |
| 12 | *Drosophila mojavensis* | DmIdgf 4 | XP_002010558.2 |
| 13 | *Drosophila erecta* | DeIdgf 4 | XP_001977287.1 |
| 14 | *Drosophila willistoni* | DwIdgf 4 | XP_002071011.1 |
| 15 | *Drosophila melanogaster* | DmIdgf 4, isoform C | NP_001285068.1 |
| 16 | *Drosophila suzukii* | DsIdgf 4 | XP_016937309.1 |
| 17 | *Drosophila busckii* | DbIdgf 4 | XP_017853261.1 |
| 18 | *Drosophila ananassae* | DaIdgf 4 | XP_001966699.3 |
| 19 | *Drosophila hydei* | DhIdgf 4 | XP_023176692.1 |
| 20 | *Zeugodacus cucurbitae* | **ZcIDGF 6** | XP_011187150.1 |
| 21 | *Bactrocera dorsalis* | BdIdgf 6 | AQR60115.1 |
| 22 | *Bactrocera oleae* | BoIdgf 6 isoform X1 | XP_014100338.1 |
| 23 | *Rhagoletis pomonella* | RpIdgf 6 | XP_036345932.1 |
| 24 | *Bactrocera oleae* | BoIdgf 6 isoform X2 | XP_036231254.1 |
| 25 | *Scaptodrosophila lebanonensis* | SlIdgf 6 | XP_030388359.1 |
| 26 | *Drosophila melanogaster* | DmIdgf 6 isoform B | NP_001286499.1 |
| 27 | *Drosophila sechellia* | DsIdgf 6 | XP_032572991.1 |
| 28 | *Drosophila mauritiana* | DmIdgf 6 | XP_033156614.1 |
| 29 | *Drosophila busckii* | DbIdgf 6 | XP_017838358.1 |
| 30 | *Drosophila erecta* | DeIdgf 6 | XP_001975322.1 |
| 31 | *Drosophila virilis* | DvIdgf 6 | XP_002049011.1 |
| 32 | *Drosophila pseudoobscura* | DpIdgf 6 | XP_001362042.2 |
| 33 | *Drosophila novamexicana* | DnIdgf 6 | XP_030566477.1 |
| 34 | *Drosophila innubila* | DiIdgf 6 | XP_034477457.1 |
| 35 | *Drosophila hydei* | DhIdgf 6 | XP_023163971.2 |
| 36 | *Drosophila albomicans* | DaIdgf 6 | XP_034111086.1 |
| 37 | *Drosophila guanche* | DgIdgf 6 | XP_034120461.1 |
| 38 | *Zeugodacus cucurbitae* | ZcIDGF 4_1 | XP_011189060.1 |
| 39 | *Bactrocera oleae* | BoIdgf 5 | XP_014085185.1 |
| 40 | *Bactrocera latifrons* | BlIdgf 5 | XP_018798938.1 |
| 41 | *Ceratitis capitata* | CcIdgf 4 | XP_004520935.3 |
| 42 | *Rhagoletis zephyria* | RzIdgf 5 isoform X1 | XP_017478398.1 |
| 43 | *Rhagoletis pomonella* | RpIdgf 5 | XP_036330312.1 |
| 44 | *Drosophila arizonae* | DaIdgf 4 isoform X2 | XP_017872390.1 |
| 45 | *Drosophila virilis* | DvIdgf 4 | XP_002055558.2 |
| 46 | *Drosophila virilis* | DvIdgf 5 | XP_002050991.1 |
| 47 | *Drosophila novamexicana* | DnIdgf 5 | XP_030558187.1 |
| 48 | *Scaptodrosophila lebanonensis* | SlIdgf 5 | XP_030381285.1 |
| 49 | *Drosophila innubila* | DiIdgf 4 | XP_034474469.1 |
| 50 | *Rhagoletis pomonella* | RpIdgf 4 | XP_036318935.1 |
| 51 | *Drosophila arizonae* | DaIdgf 5 | XP_017865855.1 |
| 52 | *Rhagoletis zephyria* | RzIdgf 4 | XP_017484636.1 |
| 53 | *Drosophila grimshawi* | DgIdgf 5 | XP_001986987.1 |
| 54 | *Drosophila melanogaster* | DmIdgf 5 | NP_611321.3 |
| 55 | *Zeugodacus cucurbitae* | ZcIDGF 3_1 | XP_028898008.1 |
| 56 | *Bactrocera oleae* | XP_036222922.1 | XP_036222922.1 |
| 57 | *Drosophila busckii* | XP_017854707.2 | XP_017854707.2 |
| 58 | *Drosophila obscura* | XP_022214675.1 | XP_022214675.1 |
| 59 | *Drosophila bipectinata* | XP_017105054.1 | XP_017105054.1 |
| 60 | *Drosophila erecta* | XP_001976221.2 | XP_001976221.2 |
| 61 | *Drosophila ananassae* | XP_001963329.2 | XP_001963329.2 |
| 62 | *Bactrocera dorsalis* | XP_011206953.1 | XP_011206953.1 |
| 63 | *Bactrocera dorsalis* | BdIdgf 2 | XP_011206954.1 |
| 64 | *Ceratitis capitata* | CcIdgf 2 | XP_004518572.1 |
| 65 | *Rhagoletis pomonella* | RpIdgf 2 isoform X2 | XP_036329035.1 |
| 66 | *Bactrocera dorsalis* | BdIdgf 3 | AUP42572.1 |
| 67 | *Rhagoletis pomonella* | RpIdgf 3 isoform X1 | XP_036329034.1 |
| 68 | *Ceratitis capitata* | CcIdgf 3 | XP_004518571.1 |
| 69 | *Rhagoletis pomonella* | RpIdgf3 isoform X3 | XP_036329036.1 |
| 70 | *Rhagoletis pomonella* | RpIdgf 2 | XP_036329940.1 |
| 71 | *Rhagoletis pomonella* | RpIdgf 3 | XP_036329939.1 |
| 72 | *Drosophila mojavensis* | DmIdgf 2 | XP_002002216.1 |
| 73 | *Drosophila hydei* | DhIdgf 2 | XP_023163128.2 |
| 74 | *Drosophila novamexicana* | DmIdgf 2 isoform X1 | XP_030557217.1 |
| 75 | *Drosophila navojoa* | DnIdgf 2 | XP_030241800.1 |
| 76 | *Scaptodrosophila lebanonensis* | SlIdgf 2 | XP_030384792.1 |
| 77 | *Drosophila pseudoobscura* | DpIdgf 2 | XP_001356912.1 |
| 78 | *Drosophila virilis* | DvIdgf 2 | XP_002052256.1 |
| 79 | *Zeugodacus cucurbitae* | ZcIDGF 1 | XP_011187129.1 |
| 80 | *Bactrocera latifrons* | BlIdgf 1 | XP_018791184.1 |
| 81 | *Bactrocera dorsalis* | BdIdgf 1 | XP_011206956.1 |
| 82 | *Bactrocera oleae* | BoIdgf 1 | XP_014103107.1 |
| 83 | *Ceratitis capitata* | CcIdgf 1 | XP_004518573.1 |
| 84 | *Rhagoletis pomonella* | RpIdgf 1 | XP_036329037.1 |
| 85 | *Rhagoletis zephyria* | RzIdgf 1 | XP_017464943.1 |
| 86 | *Scaptodrosophila lebanonensis* | SlIdgf 1 | XP_030384793.1 |
| 87 | *Drosophila novamexicana* | DnIdgf 1 | XP_030557216.1 |
| 88 | *Drosophila biarmipes* | DbIdgf 1 | XP_016964824.1 |
| 89 | *Drosophila virilis* | DvIdgf 1 | XP_002052257.1 |
| 90 | *Drosophila simulans* | DsIdgf 1 | XP_002079638.2 |
| 91 | *Drosophila yakuba* | DyIdgf 1 | XP_002090419.1 |
| 92 | *Drosophila miranda* | DmIdgf 1 | XP_017153917.1 |
| 93 | *Drosophila willistoni* | DwIdgf 1 | XP_002066870.1 |
| 94 | *Drosophila persimilis* | DpIdgf 1 | XP_002015319.1 |
| 95 | *Drosophila guanche* | DgIdgf 1 | XP_034126465.1 |
| 96 | *Drosophila erecta* | DeIdgf 1 | XP_001976219.1 |
| 97 | *Drosophila ananassae* | DaIdgf 1 | XP_001963330.1 |

# Figure S1: Predicted N-glycosylation sites in amino acid sequences of IDGFs using online NetNGlyc 1.0 Server (http://www.cbs.dtu.dk/services/NetNGlyc/).


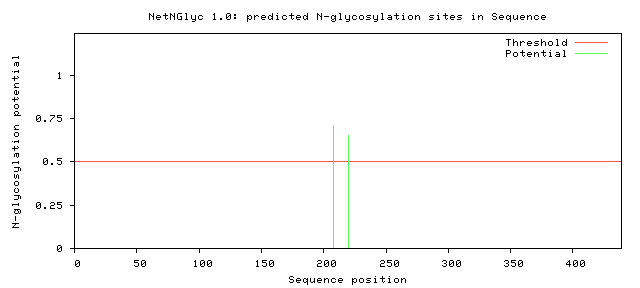


A: Predicted N-glycosylation sites in amino acid sequence of *IDGF1.*


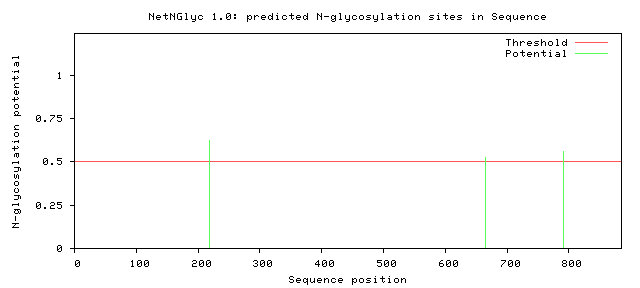


B: Predicted N-glycosylation sites in amino acid sequence of *IDGF3_1*.


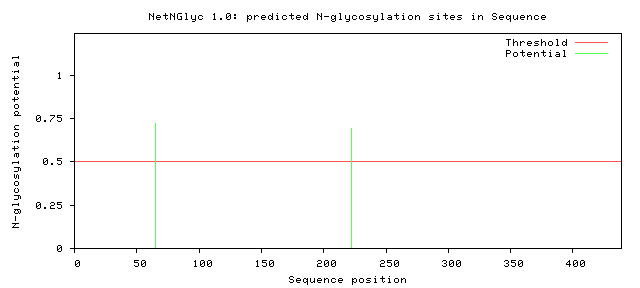


C: Predicted N-glycosylation sites in amino acid sequence of *IDGF4_0*.


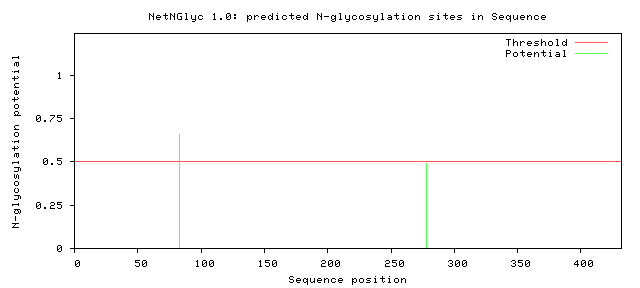


D: Predicted N-glycosylation sites in amino acid sequence of *IDGF4_1*.


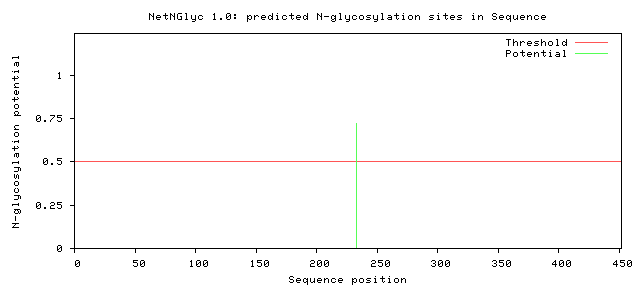


E: Predicted N-glycosylation sites in amino acid sequence of *IDGF6*.

**Table S2: Characteristics of 5 chitinase genes of *Zeugodacus cucurbitae.***

| **Gene name** | **Product size (bp)** | **Amino acid** | **Molecular weight** | **Isoelectric point** | **Gene bank accession no.** |
| --- | --- | --- | --- | --- | --- |
| *IDGF1* | 1543 | 439 | 49.42kDa | 5.99 | [NW_011863743](https://www.ncbi.nlm.nih.gov/nuccore/NW_011863743) |
| *IDGF3_1* | 3018 | 885 | 98.95kDa | 6.06 | [NW_011863743](https://www.ncbi.nlm.nih.gov/nuccore/NW_011863743) |
| *IDGF4_0* | 1668 | 432 | 48.34kDa | 5.768 | [NW_011863773](https://www.ncbi.nlm.nih.gov/nuccore/NW_011863773) |
| *IDGF4_1* | 1402 | 439 | 48.45kDa | 6.766 | [NW_011863723](https://www.ncbi.nlm.nih.gov/nuccore/NW_011863723) |
| *IDGF6* | 1718 | 452 | 50.35kDa | 6.682 | [NW_011863744](https://www.ncbi.nlm.nih.gov/nuccore/NW_011863744) |

**Table S3: Primers used for cloning, dsRNA and real time qRT-PCR amplification.**

| **Gene** | **Primer** | **Sequence** | **Size** |
| --- | --- | --- | --- |
| ***IDGF1*** | whole seq-F  whole seq-R  dsRNA –F  dsRNA –R  qpcr-F  qpcr-R | CCAAGCAGCGTTCGTACAAC  CTTATAAGAATTGCTTAATCGCACG  GGATCCTAATACGACTCACTATAGGCCAAGCAGCGTTCGTACAAC  GGATCCTAATACGACTCACTATAGGGATTCGCTTCGTCCACATCAC  CTTTCGTTGGCGTTGACC  GGCTTCGTCTGGATTACG | 1388  407  138 |
| ***IDGF3_1*** | whole seq-F  whole seq-R  dsRNA –F  dsRNA –R  qpcr-F  qpcr-R | CGTCTACTAAAAGCGCTATC  CTCGAACATTACCTTTAGCTTCCAC  GGATCCTAATACGACTCACTATAGGCGTCTACTAAAAGCGCTATC  GGATCCTAATACGACTCACTATAGGCCCTCCAACAGTTCGAGATAC  GCCTTCGATTTCCTCACAC  CACGTTGCAACAACCAATTC | 2836  440  136 |
| ***IDGF4_0*** | whole seq-F  whole seq-R  dsRNA –F  dsRNA –R  qpcr-F  qpcr-R | GGCGCACCGGTTTTAACTTAAACC  CATGCTCCGAACAATGAGAC  GGATCCTAATACGACTCACTATAGGCGAGCAACTCACACCTTG  GGATCCTAATACGACTCACTATAGGCGAAACCATACGTCTTCACC  GACTTTGTTGTGGATGAGAAGG  GCATGCAAGTTGACGTAATCC | 1594  381  188 |
| ***IDGF4_1*** | whole seq-F  whole seq-R  dsRNA –F  dsRNA –R  qpcr-F  qpcr-R | CGAACCGTGCAGTACATTTTG  CAAAGAACTGGTTTTACAATTTG  GGATCCTAATACGACTCACTATAGGCGAACCGTGCAGTACATTTTG  GGATCCTAATACGACTCACTATAGGGATTCTCCAGCACGTTGAGG  GGCATGGCAGTTTCCTAAAA  TCTGCAGCGTTTTCATCAAC | 1349  401  117 |
| ***IDGF6*** | whole seq-F  whole seq-R  dsRNA –F  dsRNA –R  qpcr-F  qpcr-R | GCTGCGATCAGTTTCAGTTTG  CTTGGCAATAAGTGGAAGC  GGATCCTAATACGACTCACTATAGGCTCGAATTGTTGGAGAACCC  GGATCCTAATACGACTCACTATAGGGATCGGCCAATTCGGGATTG  CCAACAAAGTGGGCAATC  CCATAGACAAGGTAGTCGC | 1705  412  151 |
| ***EFα1-rt*** | qpcr-F  qpcr-R | CGTTGGTGTCAACAAGATGG  TGCCTTCAGCATTACCTTCC | 230 |
| ***GFP*** | dsRNA-F  dsRNA-R  RT-PCR-F  RT-PCR-R | TAATACGACTCACTATAGGGCAGTGGAGAGGGTGAA  TAATACGACTCACTATAGGGTTGACGAGGGTGTCTC  CAGTGGAGAGGGTGAAG  TTGACGAGGGTGTCTC | 711 |


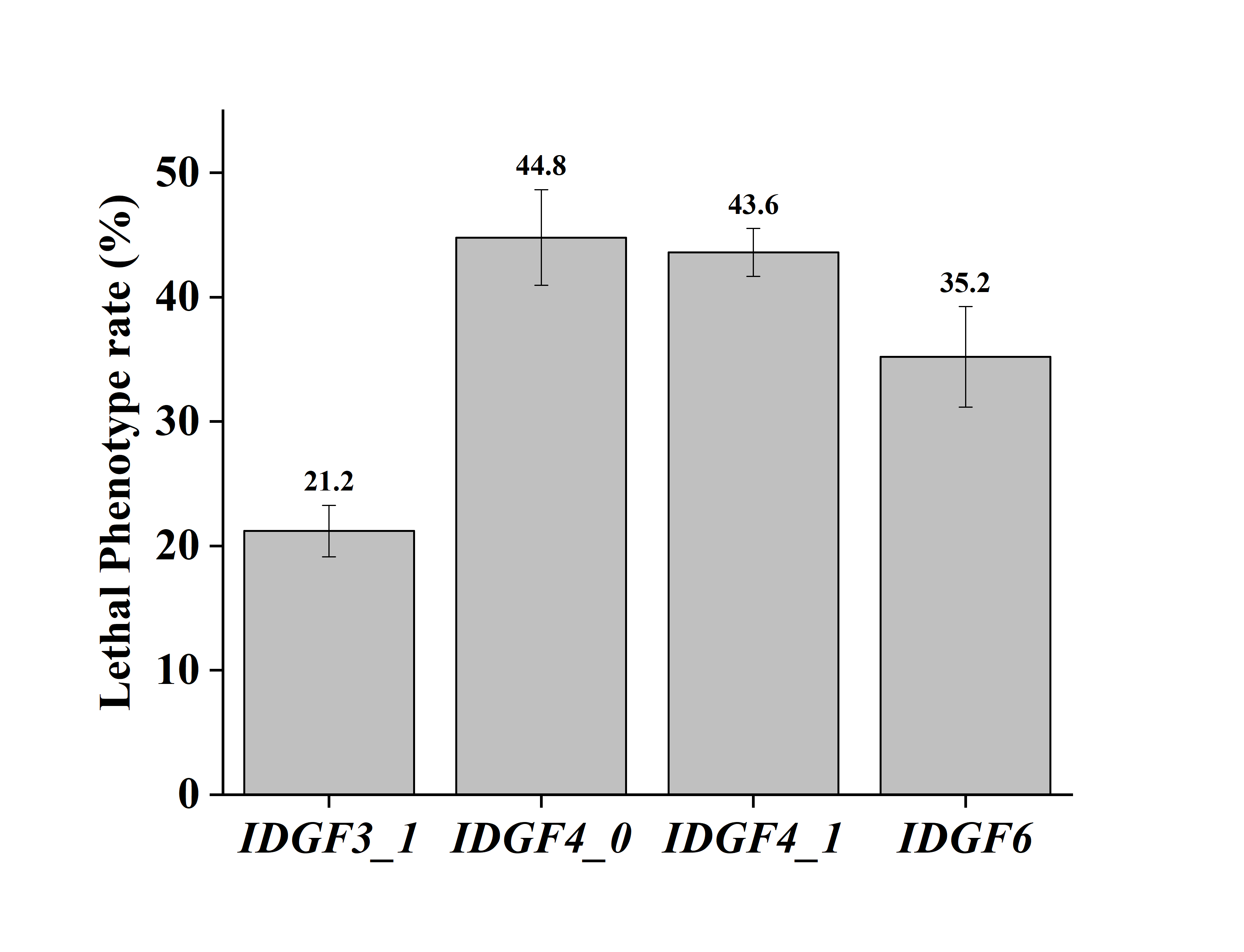


**Figure S2: Percentage of positive phenotype post feeding dsRNA of IDGFs in *Z. cucurbitae*.**
